# Cortical Thinning and Abnormal Structural Covariance Network After Three Hours Sleep Restriction

**DOI:** 10.3389/fpsyt.2021.664811

**Published:** 2021-07-20

**Authors:** Chaoyan Wang, Peng Zhang, Caihong Wang, Lu Yang, Xinzhong Zhang

**Affiliations:** Key Laboratory of Neurorestoratology, The First Affiliated Hospital of Xinxiang Medical University, Henan, China

**Keywords:** neuroimaging, cortical thickness, structural covariance network, graph theory, sleep restriction

## Abstract

Sleep loss leads to serious health problems, impaired attention, and emotional processing. It has been suggested that the abnormal neurobehavioral performance after sleep deprivation was involved in dysfunction of specific functional connectivity between brain areas. However, to the best of our knowledge, there was no study investigating the structural connectivity mechanisms underlying the dysfunction at network level. Surface morphological analysis and graph theoretical analysis were employed to investigate changes in cortical thickness following 3 h sleep restriction, and test whether the topological properties of structural covariance network was affected by sleep restriction. We found that sleep restriction significantly decreased cortical thickness in the right parieto-occipital cortex (Brodmann area 19). In addition, graph theoretical analysis revealed significantly enhanced global properties of structural covariance network including clustering coefficient and local efficiency, and increased nodal properties of the left insula cortex including nodal efficiency and betweenness, after 3 h sleep restriction. These results provided insights into understanding structural mechanisms of dysfunction of large-scale functional networks after sleep restriction.

## Introduction

Sleep loss is becoming a common and serious issue nowadays. Insufficient sleep (e.g., <7 h) can lead to cumulative deterioration in neurobehavioral performance, resulting in destabilized wake state, impaired cognition, and behavior (1, 2). Additionally, cumulative sleep loss is associated with sleep disorders (3), which incurs considerable social and health-related costs. Thus, elucidating the mechanisms underlying sleep loss is an important goal in basic and clinical neuroscience.

It has been suggested that acute sleep deprivation is involved in impaired attention, emotion discrimination, and expression (4). For example, participants with insufficient sleep had impaired capacity to sustain attention over time and usually rated neutral images as more emotionally negative (4, 5). Sleep deprivation resulted in altered activation in the dorsolateral prefrontal cortex, intraparietal sulcus, amygdala, and insula cortex (5–7), as well as abnormal functional connectivity between distributed brain areas, such as the insula connectivity to prefrontal cortex (8), hippocampal connectivity to thalamus, motor, and visual-related regions (9, 10). Additionally, graph theoretical analysis on functional connectivity network revealed that 34 h of sleep deprivation was related with enhanced small-world property, global, and local efficiency (11). Although sleep deprivation altered large-scale functional networks, the structural network substrates underlying the dysfunction remain unclear.

Structural neuroimaging studies suggested that insufficient sleep was also associated with brain structural abnormalities. A voxel-based morphometry study found that gray matter volume of the ventromedial prefrontal cortex was related to general daytime sleepiness (12). Altered gray matter volume of the thalamus and anterior insula, as well as cortical thinning of the bilateral parietal cortex was observed after sleep deprivation (13–15). Furthermore, morphological alteration was negatively associated with sleepiness. It has been well-recognized that structural changes of one brain region often co-vary with the changes in another brain area (16, 17). For example, individuals with greater cortical thickness of Broca's area have also greater cortical thickness of Wernicke's area (18). This phenomenon is known as structural covariance, which has been demonstrated to be abnormal in several neuropsychiatric disorders (19–21). Given that sleep deprivation altered the morphology of specific brain areas, we speculated that the structural covariance network might be also influenced by sleep deprivation.

Additionally, it is well known that the sleep deprivation-related brain functional and structural changes were age dependent. A verbal encoding task study observed sleep-related high and low activation of anterior hippocampus in old individuals and young individuals, respectively (22). Functional and structural magnetic resonance imaging (MRI) studies found altered resting-state functional connectivity and gray matter volume of anterior insula after sleep deprivation only in young adults (8, 15). Only young adults showed correlation of medial temporal lobe network connectivity with sleep quality (23). These findings might imply that young people and old people have different brain mechanism of sleep deprivation.

A previous study found cortical thinning after long-term 23-h sleep deprivation (14). However, whether the cortical thinning was observed following 3-h sleep restriction remains unclear. Thus, the aims of current study were to (1) investigate whether cortical thickness was affected by 3-h sleep restriction, (2) investigate sleep restriction-related structural covariance network abnormalities by employing graph theory, and (3) test whether those alterations were age dependent.

## Materials and Methods

### Study Design and Participants

The database employed in the current study was obtained from the Sleepy Brain Project (https://openneuro.org/datasets/ds000201). Eighty healthy participants including 37 old adults (65–75 years, mean age: 68.8 years) and 43 young adults (20–30 years, mean age: 23.5 years) in total were included. Participants were screened using the following inclusion criteria: no ferromagnetic items in body, not claustrophobic, not pregnant, not color blind, no shift work, have no current or past psychiatric or neurological illness (including addiction), do not have hypertension or diabetes, and do not use psychoactive or immune-modulating drugs.

This study had a crossover within-group design. The procedures of study design were as follows: All participants were randomized to undergo both conditions of one night sleep restriction (sleep only 3 h) and full sleep (normal sleep) in a counterbalanced order with an interval of 1 month between sessions. Sleep diaries were recorded three nights before the experiment. Participants were told to avoid coffee, alcohol, and taking naps on the experimental day. On the night before imaging, participants slept in their own homes. Sleep was monitored using ambulatory polysomnography in the homes of participants. Standard electrode montage for electroencephalogram (EEG) sleep recording was used (C3, C4 referenced to the contralateral mastoid). Additionally, two submental electrodes were used for electromyography. One electrode at each of the outer canthi of the eyes was used for electrooculography. In the sleep restriction condition, participants were instructed to go to bed 3 h before the time they would usually get up and then get up at their normal time. Magnetic resonance imaging was performed in the evening following sleep restriction or normal sleep. After scanning, participants completed several behavior questionnaires, including Hospital Anxiety and Depression scale (HADS, characterizing participants' anxiety and depression severity) (24), Epworth Sleepiness Scale (ESS, characterizing participants' sleepiness) (25), and KSQ sleep quality index and snoring symptom index (characterizing participants' sleep quality and snoring symptom) (26).

The Sleepy Brain Project was preregistered at clinicaltrials.gov (https://clinicaltrials.gov/ct2/show/NCT02000076). This study was approved by the Regional Ethics Review Board of Stockholm (2012/1870–32). All participants provided written informed consent before participating in the current study. All experiments were performed in accordance with the Declaration of Helsinki and applicable local regulations. Detailed demographic information of participants can be found in Table 1.

**Table 1:** Demographic information of subjects in the old group and young group.

	**Young group**	**Old group**	***p*-Value**
	**(*n* = 43)**	**(*n* = 37)**	
Gender (female/male)	21/22	20/17	0.64^a^
HADS anxiety score (mean ± SD)	2.79 ± 2.45	1.46 ± 1.46	**0.005^b^**
HADS depression score (mean ± SD)	1.09 ± 1.31	1.14 ± 0.98	0.87^b^
ESS (mean ± SD)	7.23 ± 3.01	8.65 ± 4.8	0.11^b^
KSQ sleep quality index (mean ± SD)	5.26 ± 0.43	5.19 ± 0.48	0.52^b^
KSQ snoring symptom index (mean ± SD)	5.88 ± 0.32	5.65 ± 0.54	**0.02^b^**

a *Chi-square test*.

b *Two-tailed two sample t-test*.

*Bold p-values indicate significant difference in sleep measures between young group and old group. SD, standard deviation; HADS, Hospital Anxiety and Depression scale; ESS, Epworth Sleepiness Scale; KSQ, Karolinska Sleep Questionnaire*.

### Scan Acquisition

The MRI data of the Sleepy Brain Project were acquired using a General Electric Discovery 3T MRI scanner. Echo-planar images were obtained using the following parameters: flip angle 75°, TE 30, TR 2.5 s, field of view 28.8 cm, slice thickness 3 mm, 49 slices. The T1 structural image was scanned using a sagittal BRAVO sequence, 24 cm field of view, and 1 mm slice thickness. Other parameters can be seen in previous studies (27, 28).

### Preprocessing

The T1 images were automatically preprocessed using Freesurfer software version 6.0 (https://surfer.nmr.mgh.harvard.edu/fswiki). Briefly, following intensity normalization, non-brain tissues were removed from individual T1 images using a hybrid watershed/surface deformation procedure (29). The gray matter and white matter were segmented and then used for cortical reconstruction. The white surface (defined as gray/white boundary) and pial surface (defined as gray/CSF boundary) were then identified. Cortical thickness was calculated as the closest distance from the gray/white boundary to the gray/CSF boundary at each vertex. In order to increase sensitivity to within-subject variability, a longitudinal processing procedure was employed. First, datasets from each time point were processed independently as mentioned above. Second, an unbiased subject-specific template was obtained by combining data from the two time points for each subject, avoiding possible asymmetries or biases (30). Finally, each time point was aligned with the subject-specific unbiased template, resulting in 160 datasets. All datasets from each step were inspected visually. Manual editing was conducted if necessary.

The individual maps of cortical thickness cannot be compared because they have a different number of vertex. Thus, those maps were then warped and registered to an average spherical space (fsaverage) that optimally aligned sulcus and gyrus patterns, thus, enabling matching of cortical locations among individuals across the whole surface. The registered cortical thickness maps in *f* 's average space were obtained for each hemisphere and smoothed with 10-mm full width at half maximum for statistical analysis.

### Statistical Analysis

The difference in demographic information between young adults and old adults were tested using chi-square test or two-tailed two-sample *t*-test in SPSS. Statistical level of *p* < 0.05 was considered as significant.

Two-way mixed analysis of variance (between-subject factor: age; within-subject factor: deprivation) was employed to investigate age effect on changes of cortical thickness after sleep restriction. The multiple comparisons were corrected by using Z Monte Carlo simulations (31) at cluster level of 0.05. The cluster-forming threshold was set to be *p* < 0.01. For the significant main effect of deprivation, we further investigated whether cortical thickness was higher or lower in sleep restriction condition compared with full sleep condition. For the significant age × deprivation interaction effect, we separately analyzed sleep restriction-related cortical thickness changes in young adults and old adults. Multiple comparisons were corrected using Bonferroni method with *p* < 0.05/6.

Pearson correlation analysis was performed to investigate correlation between changes (sleep restriction minus full sleep) in cortical thickness of those brain areas and individual characteristics including HADS scores, ESS score, KSQ sleep quality index, and snoring symptom index. Statistical level of *p* < 0.05 was considered significant.

### Structural Covariance Network Analysis

The procedures mentioned above only tell us which single brain areas were affected by sleep restriction. In order to investigate whether sleep restriction had influence on structural connectivity between brain areas, we conducted structural covariance network analysis. Structural covariance network was constructed across all participants. First, the Desikan–Killiany cortical atlas was employed to construct cortical thickness-based correlation matrix for sleep restriction condition and full sleep condition by employing Pearson correlation analysis. Those correlation matrices were thresholded by using false discovery rate (FDR) method with *q* < 0.05, in order to remove weak and spurious correlations. The correlation value was set to be one if it was higher than the threshold, and zero otherwise. Considering that it is difficult to interpret the negative correlation, so we excluded the negative correlation value in the current study, as suggested by previous studies (32, 33). Global and local network properties including small worldness, global/local efficiency, betweenness, nodal degree, and nodal efficiency were then computed as follows.

Small-world properties were originally proposed by Watts and Strogatz (34). The clustering coefficient of a node was defined as the likelihood whether its neighborhoods were connected with each other or not. The overall clustering coefficient was computed as the average of clustering coefficient across all nodes in the network. Clustering coefficient is a measure of the extent of local information communication of the network. The path length is the number of edges included in the path connecting two nodes. The shortest path length of a node was defined as the averaged shortest path length between the node and all other nodes. The mean shortest path length of a network was then the average of the shortest path lengths across all nodes. The shortest path length quantifies the ability for information propagation in parallel (35).

A real network would be considered small world if it has similar shortest path length but higher clustering coefficient than random network, that is, γ = *C*_*real*_/*C*_*rand*_ > 1, λ = *C*_*real*_/*C*_*rand*_ ≈ 1 (34), where *C*_*real*_ and *C*_*real*_ are the clustering coefficient and shortest path length of real network, *C*_*rand*_ and *C*_*rand*_ are the clustering coefficient and shortest path length of 100 matched random networks that preserve the same number of nodes, edges, and degree distribution as the real network (36). These two measurements can be summarized into a scalar quantitative metric, small worldness, σ = γ/λ, which is typically >1 in the case of small-world organization. A network that had high small worldness value often had high efficiency of information communication and high local clustering.

The global efficiency of a network is defined by the inverse of the harmonic mean of the minimum path length between each pair of nodes, which is a measure of parallel information transformation. The local efficiency of a network is the averaged local efficiency of each node, which is defined as the global efficiency of sub-graph of the node. The local efficiency can be understood as a measure of fault tolerance of the network, indicating how well each sub-graph exchanges information when the index node is eliminated.

To determine the nodal properties of structural covariance network, we computed the nodal degree, nodal efficiency, and betweenness. The nodal degree of a given node was defined as the sum of the edges that connected to the node. The nodal efficiency of a node is computed as the inverse of the harmonic mean of the minimum path length between the node and all other nodes, which measures the information propagation ability of a node with the rest of the nodes in the network. The betweenness of a node is measured as the fraction of all shortest paths in the network that pass through the node. Betweenness captures the influence of a node over information flow between all other nodes in the network. Notably, these nodal properties reflect the importance of a node in a network from different aspects (37, 38). For example, nodes with high degree can be considered as centers for information integration; those with high efficiency are relevant for information flow; those with high betweenness may serve as way station for information communication.

The statistical differences in network properties between sleep restriction condition and full sleep condition were investigated by using non-parametric permutation test (1,000 permutations). For each permutation and each individual's paired data, we randomly assigned one to sleep restriction condition and the other one to full sleep condition. The structural covariance networks were then constructed for both conditions, followed by computing the network properties. A null distribution of difference in network properties was then obtained. A *p*-value was calculated for each network property by computing the proportion of difference exceeding the null distribution. For global properties including clustering coefficient, shortest path length, γ, λ, α, global and local efficiency, a *p*-value of <0.05 was considered significant. For nodal properties including nodal degree, nodal efficiency, and betweenness, an FDR method with *q* < 0.05 was employed to correct multiple comparisons across nodes.

In order to investigate age effect on sleep restriction-related changes of structural covariance network properties, we separately performed the structural covariance network analysis in young adults and old adults. The procedures were the same as that mentioned above.

## Results

There was no significant difference (*p* > 0.05) in gender, HADS depression score, ESS score, and KSQ sleep quality index between young adults and old adults. Young adults had significant higher HADS anxiety score and KSQ snoring symptom index compared with old adults (Table 1). The sleep diaries showed that young participants and old participants had normal sleep times of 8.46 ± 0.79 and 8.34 ± 0.85 h, respectively. Ambulatory polysomnography data showed that the total sleep time in the sleep restriction condition was 2.97 ± 0.59 and 2.52 ± 0.4 h for young adults and old adults, separately. While in the normal sleep condition, the total sleep time was 6.71 ± 1.48 and 5.56 ± 1.34 h for the young group and old group.

**Figure 1:**
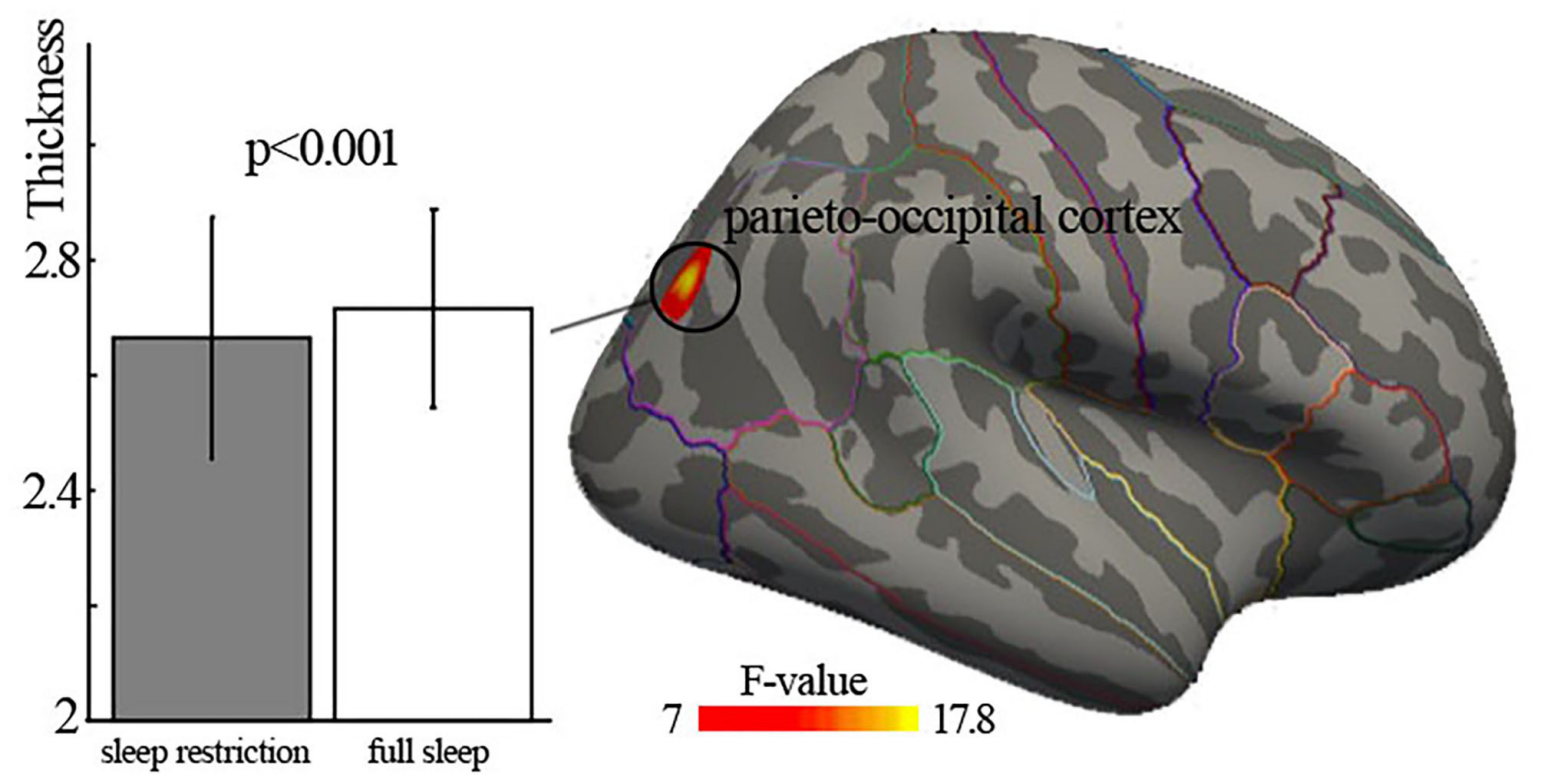
Significant main effect of deprivation on cortical thickness. Significant cortical thinning of right parieto-occipital cortex (Brodmann area 19) was found. The lines on the surface are the borders of regions of interest in Desikan–Killiany cortical atlas.

We found significantly the main effect of deprivation in the right parieto-occipital cortex (Brodmann area 19), with decreased cortical thickness in sleep restriction condition compared with full sleep condition (Figure 1, Table 2). There was no significant age × deprivation interaction effect (*p* > 0.05). There was also no significant correlation (*p* > 0.05) between cortical thickness changes of the right parieto-occipital cortex and HADS scores, ESS, KSQ sleep quality index, and snoring symptom index.

**Table 2:** Significantly cortical thickness changes after 3-h sleep restriction.

**Brain area**	**Brain size (mm^**2**^)**	**Number of vertex**	**MNI coordinate**			**Peak value**
			**x**	**y**	**z**	
Right parieto-occipital cortex	358	524	38	−76	30	−3.95

**Figure 2:**
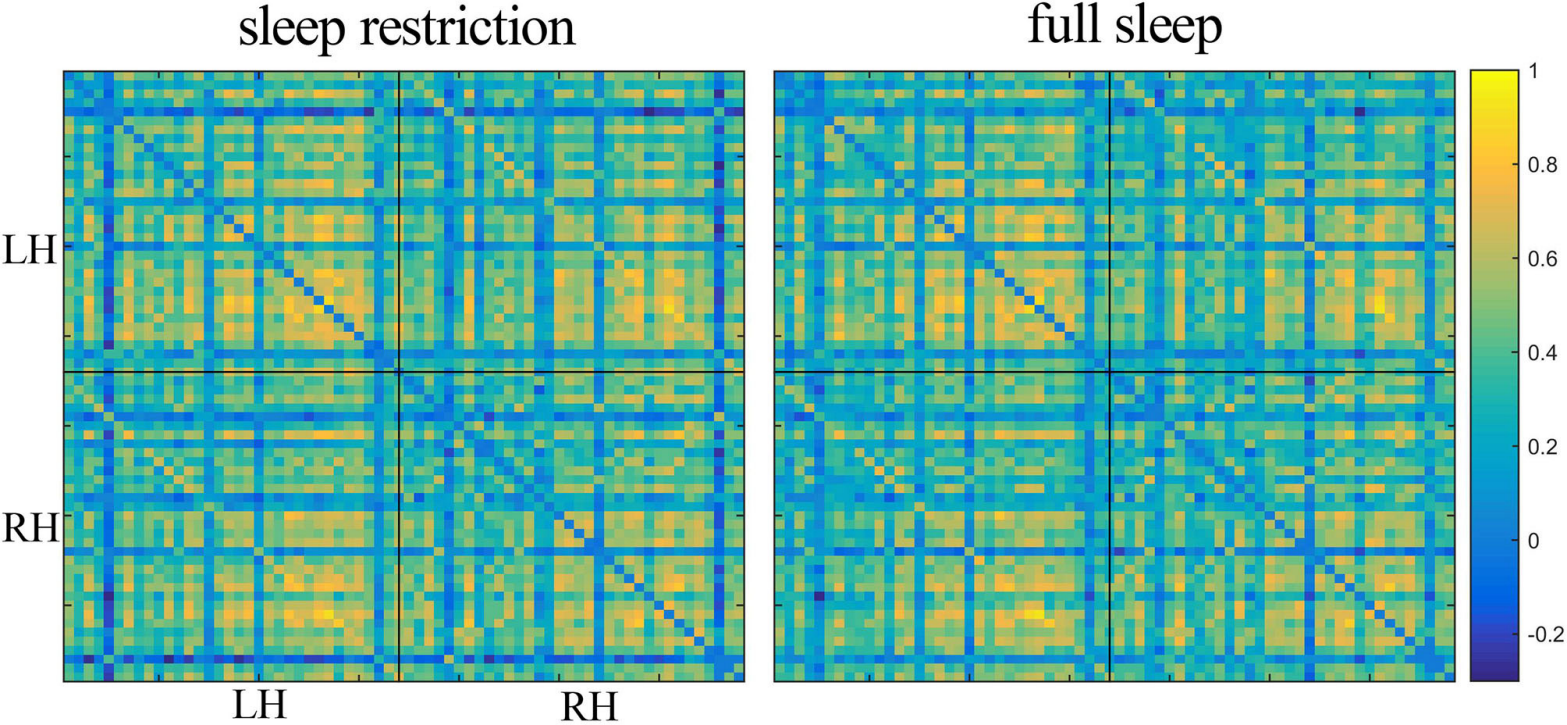
Structural covariance connectivity matrix in the sleep restriction condition **(left)** and full sleep condition **(right)**. The color bar denotes Pearson correlation values with warm colors indicating positive correlation, and blue colors indicating negative correlation. Both conditions had similar covariance connectivity pattern. LH, left hemisphere; RH, right hemisphere.

**Figure 3:**
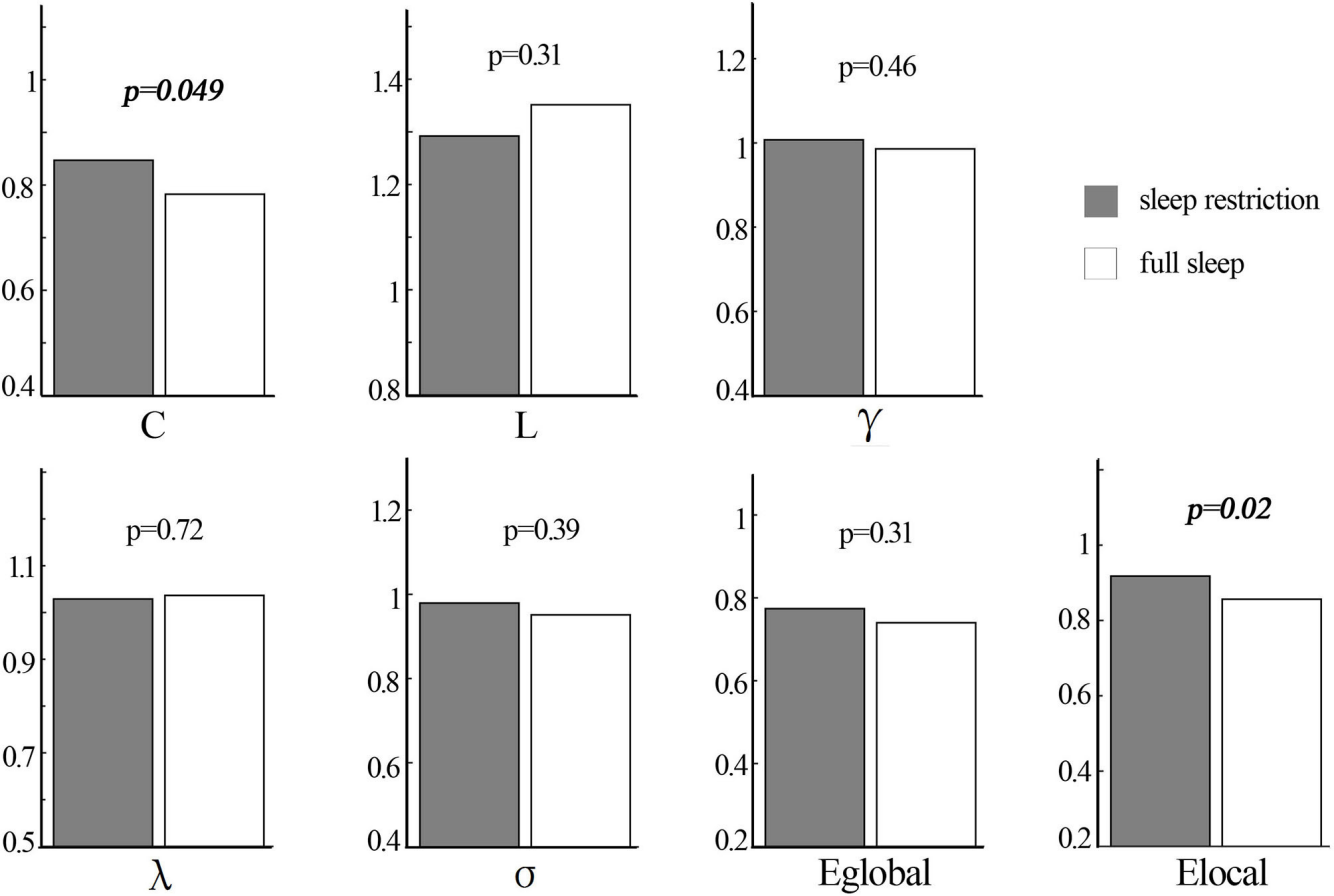
Statistical difference in topological properties of structural covariance network between sleep restriction condition and full sleep condition by using non-parametric permutation test. The clustering coefficient and local efficiency were significantly increased after sleep restriction. C, clustering coefficient; L, shortest path length; Eglobal, global efficiency; Elocal, local efficiency.

**Figure 4:**
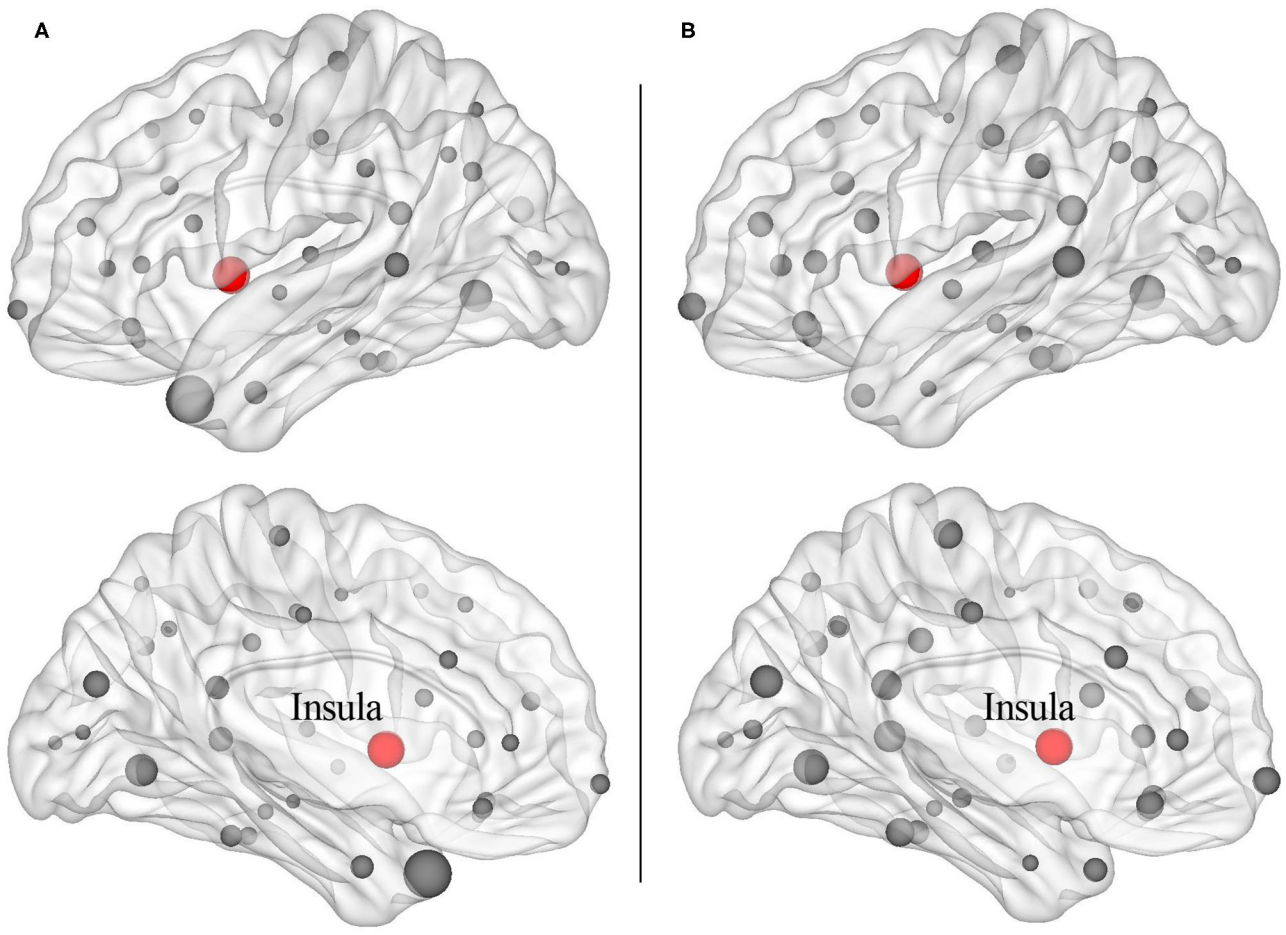
Increased nodal properties including betweenness **(A)** and nodal efficiency **(B)** after 3 h sleep restriction. The balls with red color were left insula cortex showing enhanced nodal properties after sleep restriction (false discovery rate, *q* < 0.05).

The structural covariance networks of sleep restriction condition and full sleep condition can be found in Figure 2. Both conditions showed similar structural covariance connectivity patterns. The strongest connectivity values were in the prefrontal cortex. Graph theoretical analysis revealed that clustering coefficient and local efficiency of structural covariance network were significantly (*p* < 0.05) increased after sleep restriction (Figure 3). There were no significant (*p* > 0.05) changes in the shortest path length, γ, λ, α, and global efficiency. In addition, we observed significant (FDR correction, *q* < 0.05) increased betweenness and nodal efficiency of left insula after sleep restriction (Figure 4). No sleep restriction-related changes in structural covariance network properties were observed in young adults and old adults.

## Discussion

In the current study, decreased cortical thickness of the right parieto-occipital cortex was observed after 3-h sleep restriction. Additionally, graph theoretical analysis of structural covariance network revealed significant sleep restriction-related enhancement of clustering coefficient and local efficiency. Moreover, we found significant increased betweenness and nodal efficiency of left insula cortex after sleep restriction. Those findings of structural changes was in line with previous long-term sleep deprivation studies (13, 14, 39), suggesting the sensitivity of brain structure to sleep deprivation.

We first observed decreased cortical thickness after 3-h sleep restriction in the right parieto-occipital cortex (Brodmann area 19). An EEG study found that parieto-occipital regions had high slow-wave activity response to sleep deprivation (40). Individuals suffering from insomnia showed decreased intrahemispheric EEG asymmetry of parieto-occipital cortex (41). A neuroimaging task study suggested that individuals with sleep deprivation had lowered neural activity of the parieto-occipital cortex, which was associated with decline in visual short-term memory (42). Those findings were consistent with the present results. In addition, this brain area belongs to the dorsal stream of the visuospatial processing, which plays an important role in retaining visual and spatial information, spatial perception, and guidance of actions (43). Sleep lose may be involved in altered visuospatial perception. For example, task studies reported that one night sleep deprivation resulted in slowing of psychomotor vigilance speed (44) and rightward shift in visuospatial judgments regarding the relative lengths of line segments (45). The results observed in the current study provided structural evidence for previous functional findings, which possibly suggested impaired function of visuospatial information processing after short-term sleep deprivation.

We did not find the relation between cortical thickness changes and sleep measures. Together with a previous study, which also found absence of the relation (15), we speculated that the sleep restriction-related structural changes are trait-like characteristics, which are independent of sleep measures. A previous study found an age × deprivation interaction effect in the gray matter volume (15). However, we did not observe the interaction effect in cortical thickness. It might be due to the fact that cortical thickness reflects only one aspect of the gray matter volume because gray matter volume is not only associated with surface area and cortical thickness but also related with the degree and pattern of folding.

Increased clustering coefficient and local efficiency after sleep restriction were observed in the current study, which was accordant with a previous functional network study reporting enhanced brain small worldness and local efficiency (11). One possible explanation for the enhancement is a compensatory adaptation of the human network under conditions of diminished processing resources due to insufficient sleep (46, 47). Another possible explanation for the overall enhancement is that, it is possibly related with increased nodal efficiency and betweenness of left insula cortex found in the current study. The insula cortex is involved in attention and interoceptive and affective processes (48). Neuroimaging studies of attention task and risk taking task both showed abnormal bilateral insula activation, indicating impaired cognitive control following sleep deprivation (49, 50). Resting-state studies reported higher amplitude of low-frequency fluctuations in the left insula and altered functional connectivity of bilateral insula with dorsal anterior cingulate cortex after sleep deprivation (51, 52). A structural study also found sleep deprivation-related gray matter volume alteration of bilateral insula (39). The increased nodal properties of the left insula observed in the current study were in line with previous reports, suggesting sleep restriction-related dysfunction of cognitive control. It is interesting that we did not observe sleep restriction-related changes in nodal properties of the parieto-occipital cortex, in which the reduction of cortical thickness was found. It may be due to the short duration of sleep restriction (3 h in the present study), which is insufficient to affect the brain areas connected to the parieto-occipital cortex.

Several limitations should be mentioned. First, we did not observe age-related effects on structural covariance network properties, which was out of our expectation. It is possibly due to the limited number of participants that were used to construct the structural covariance network in young adults and old adults. Future studies are warranted to include more participants to address the issue. Second, functional significance of the observed results is speculative, since no relation of sleep restriction-related structural changes with corresponding behavioral measure was investigated.

In conclusion, the current study found decreased cortical thickness of the right parieto-occipital cortex, enhanced overall clustering coefficient, local efficiency, and increased nodal properties of left insula following three 3-h sleep restriction. These results provided insights into understanding the structural mechanism of functional alteration at network level after sleep deprivation.

## Data Availability Statement

The datasets presented in this study can be found in online repositories. The names of the repository/repositories and accession number(s) can be found below: https://openneuro.org/datasets/ds000201.

## Ethics Statement

The studies involving human participants were reviewed and approved by the Regional Ethics Review Board of Stockholm. The patients/participants provided their written informed consent to participate in this study.

## Author Contributions

XZ designed the study. PZ, CaW, and LY collected the data and performed the data analysis. ChW drafted the manuscript. ChW, PZ, and XZ discussed and revised the manuscript. All authors contributed to the article and approved the submitted version.

## Conflict of Interest

The authors declare that the research was conducted in the absence of any commercial or financial relationships that could be construed as a potential conflict of interest.

## Abbreviations

MRI: magnetic resonance imaging
HADS: hospital anxiety and depression scale
ESS: Epworth sleepiness scale
KSQ: Karolinska sleep questionnaire
FDR: false discovery rate.
